# Meta-Analysis of the Efficacy of Ectoine Nasal Spray in Patients with Allergic Rhinoconjunctivitis

**DOI:** 10.1155/2014/292545

**Published:** 2014-05-11

**Authors:** Andrea Eichel, Andreas Bilstein, Nina Werkhäuser, Ralph Mösges

**Affiliations:** ^1^Institute of Medical Statistics, Informatics and Epidemiology, Faculty of Medicine, University of Cologne, Lindenburger Allee 42, 50931 Cologne, Germany; ^2^Bitop AG, Stockumer Straße 28, 58453 Witten, Germany

## Abstract

*Objectives.* The meta-analysis aims to investigate the efficacy of ectoine nasal spray and eye drops in the treatment of allergic rhinitis and rhinoconjunctivitis symptoms. *Design and Methods.* This meta-analysis is based on yet unpublished data of four studies. Both nasal and eye symptoms were documented in patient diary cards. All scales were transformed into a 4-point scale: 0 = no, 1 = mild, 2 = moderate, and 3 = severe symptoms. Each symptom was analysed individually in a meta-analysis of the area under the curve values as well as in a meta-analysis of pre- and posttreatment comparison. *Results.* After seven days of treatment with ectoine nasal spray both nasal and ocular symptoms decreased significantly. A strong reduction of symptom severity was shown for the parameters rhinorrhoea (31.76% reduction) and nasal obstruction (29.94% reduction). Furthermore, the meta-analyses of individual symptoms to investigate the strength of effect after seven days of medication intake showed significant improvement for nasal obstruction, rhinorrhoea, nasal itching, sneezing, itching of eyes, and redness of eyes. The improvement of the symptom nasal obstruction was associated with a strong effect 0.53 (±0.26). *Conclusions.* The ectoine nasal spray and eye drops seem to be equally effective as guideline-recommended medication in the treatment of rhinoconjunctivitis symptoms.

## 1. Introduction

Allergic rhinitis is clinically defined as an inflammation of the nose with characteristic symptoms such as rhinorrhoea, nasal obstruction, sneezing, and/or itching of the nose. The symptomatic disorder of the nasal mucosa and tissue is associated with an IgE-mediated immune response to allergens and is characterised by two phases: an immediate response after allergen exposure (early phase) and a late phase occurring up to 12 hours later, which predominantly causes nasal congestion [1]. If a concurrent respiratory infection is present, a patient's probability of developing bronchial asthma as comorbidity increases. Likewise, the risk of developing further allergies with more severe symptoms rises over the time of the disease [2].

A variety of causes for rhinitis exist in both children and adults, but 50% of all cases can be ascribed to allergy [3]. Due to its prevalence, impact on quality of life, impairment of work or school performance, reducing effect on productivity, economic burden, and risk of comorbidities, allergic rhinitis is regarded worldwide as a major chronic respiratory disease. Moreover, it can be associated with significant fatigue, mood changes, cognitive impairments, depression, and anxiety [4–8].

The optimal treatment of allergic rhinitis depends on several individual factors. A stepwise therapeutic approach, however, is generally recommended. Current guidelines favour second-generation oral or topical H1 antihistamines for treating allergic rhinitis [1, 9, 10]. Moreover, intranasal glucocorticosteroids and intranasal decongestants are highly recommended as effective treatments for nasal blockage [11].

Ectoine (2-methyl-1,4,5,6-tetrahydropyrimidine-4-carboxyclic acid) is a compatible solute which is naturally produced by bacteria, conferring resistance to external stress factors such as extreme temperatures, high salt concentrations, and ultraviolet radiation. It acts via a mechanism called “preferential exclusion” and “preferential hydration” [12]. Ectoine is expelled from proteins or lipid membranes, resulting in the modulation of the solvent characteristic of surrounding water. Thus, ectoine is able to form a protective and stabilising hydrate capsule around the protein and therefore helps to protect biomolecules and proteins from irreversible structural modifications by inhibiting dehydration. This indirect effect leads to a more compact and more stable folding of proteins and increases the stability of lipid membranes by increasing their fluidity [13]. The effect derives from the mechanism of halophilic bacteria which stabilises the osmotic balance in the microorganic cell, where extremolytes such as ectoine are accumulated in the cytosol to equal out the varying salt concentration in the outer area [14, 15]. Stabilisation of membranes such as those lining the airways or eyes might reduce the potential water loss of such membranes and protect them against invading allergens, thereby limiting the inflammatory cascade induced by stress mediators at the membrane level, as has been shown for lung epithelia and skin cells [16].* In vitro* experiments have further shown that ectoine inhibits apoptosis, triggered by nanoparticles [17], and likewise blocks the activity of ceramides, which are regarded as central molecules in the sphingolipid metabolism as well as in the induction of apoptosis [18]. Currently, ectoine is used in dermatological products for successfully treating skin diseases such as atopic dermatitis [19]. Still widely unknown is the use of ectoine in nasal sprays or eye drops. In such medical devices, ectoine may strengthen the hydroprotection of the nasal membrane and may alleviate the infection of the inflamed tissue [20].

Toxicological studies and results of human studies reflect the excellent safety profile of products containing ectoine, therewith making them promising candidates for the treatment of allergic rhinoconjunctivitis [14, 20].

With this meta-analysis we aimed to investigate the efficacy of ectoine nasal spray in the treatment of allergic rhinitis and rhinoconjunctivitis symptoms.

## 2. Material and Methods

### 2.1. Literature Search

In order to investigate the efficacy of treatment with ectoine, data from published as well as unpublished clinical studies were reviewed.

Bitop AG, a German medical device company, kindly supplied us with detailed results from several clinical and noninterventional studies launched between 2008 and 2011 investigating its allergy nasal spray based on ectoine. Additionally, we conducted a systematic and comprehensive search of scientific and medical databases for further studies and reports published until January 2013. For this purpose, a catalogue of search criteria was generated in due consideration of the question posed by this meta-analysis. Using PubMed's MeSH database, the literature search was based on the following search criteria: “Ectoin,” “ectoine”, “(*S*)-2-Methyl-1,4,5,6-tetrahydropyrimidin-4-carbonsäure,” “C_6_H_10_N_2_O_2_”, “1,4,5,6-tetrahydro-2-methyl-4-pyrimidinecarboxylic acid,” “cryoprotective cyclic amino acid,” and “rhinitis.” Although several electronic databases were searched including* PubMed, Medline, Medpilot, Web of Science, CENTRAL*,* EMBASE, *and* Google Scholar, *no further studies on this topic were found. Given the lack of appropriate hits, no additional limits regarding language, participants, publishing date, or study phase were set.

Therefore, this meta-analysis is based on unpublished data provided by Bitop AG. The study data have not been published to date since the number of participants in each trial was too small. Nowadays, large randomised controlled trials with more than 250 patients per treatment group are usually required to be considered for publication [21, 22]. In total four studies were assessed which fulfilled the inclusion criteria described below. The paediatric, randomised controlled study had been formally approved by the respective ethical review committee, whereas no ethical approval was necessary for observational trials in Germany. In all studies, patients had to sign the informed consent form to be eligible for participation.

### 2.2. Patients and Outcome Parameters

The study population comprised both adults and children with a history of allergic rhinitis or rhinoconjunctivitis, who recorded their daily allergy symptoms for at least 7 days in a patient diary. Each symptom had to be scored numerically on a 4-point scale (0 = no symptoms, 1 = mild symptoms, 2 = moderate symptoms, and 3 = severe symptoms). In case of different scaling schemes applied in a study, scores were adapted to this 4-point scale for comparability reasons.

The primary efficacy parameter was the improvement of each individual symptom (nasal congestion, rhinorrhoea, itching of the nose, sneezing, red/watery eyes, and itching of the eyes) after 7 days of treatment. Generally, patient reported that rhinitis-related symptoms occurred in the nose, eyes, and ears/palate, whereas nasal congestion and rhinorrhoea were frequently reported as most predominant.

### 2.3. Statistical Methods

For continuous data, we calculated individual and pooled statistics as mean differences with 95% confidence intervals. The efficacy parameters for each study included in the analysis were analysed using the ANOVA model [23]. Scores for each individual symptom after 7 days of medication intake were evaluated in comparison to the baseline values at Day 1. All deviating scaling systems for rating the intensity of rhinitis symptoms were adapted to a 4-point scale. If symptoms were originally rated from 0 to 8 (0 being no symptoms and 8 being very severe symptoms) the scores were transformed according to the following scheme: 0, 1 = no symptoms; 2, 3 = mild symptoms; 4, 5 = moderate symptoms; 6, 7, 8 = severe symptoms. Likewise, 12-point scales were translated into 0, 1, 2 = no symptoms; 3, 4, 5 = mild symptoms; 6, 7, 8 = moderate symptoms; 9, 10, 11, 12 = severe symptoms. In case of missing data the last-value-carried forward method was applied. If data of Day one were not available, we used the score of the following day as baseline value. Additionally, the area under the curve (AUC) from Day 1 to Day 7 was assessed for each symptom. The AUC expresses the cumulative effect of the investigational products over the course of seven days by adding up the baseline adjusted symptom scores of each day. A noninferiority margin *δ* to ensure a clinically relevant effect was not determined, since no solid historical data were available. Thus, noninferiority was assumed, when the 95% confidence interval of the overall effect size included the neutral number “0.”

Results were displayed graphically as forest plots with associated 95% confidence intervals according to Clopper and Pearson [24]. The area of each square (point estimator for odds ratio) is proportional to the weight of the corresponding study and therefore proportional to the number of patients included as well as to the precision of the effect. Heterogeneity was assessed using *I* ^2^ statistics and the random-effect model was applied for data synthesis [25].

SPSS version 19 and Review Manager 5 (RevMan 5) were used for statistical analyses and quantitative data synthesis.

## 3. Results

### 3.1. Literature Search and Study Population

**Figure 1:**
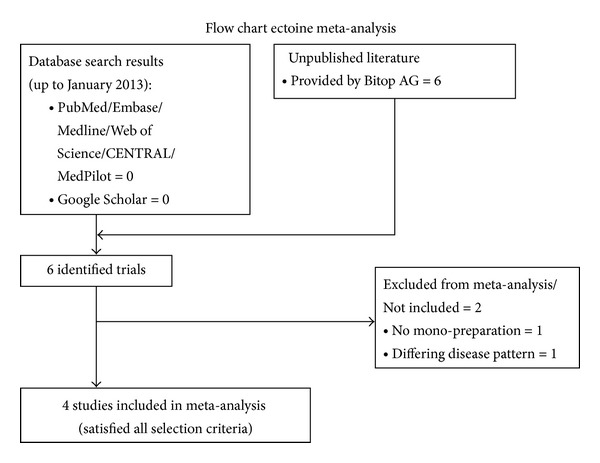
Flow chart.

We identified six studies with unpublished data, provided and conducted by Bitop AG, which matched our inclusion criteria (see Figure 1). One study investigating ectoine in combination with dexpanthenol had to be excluded, since the additional active agent dexpanthenol instead of a monopreparation would have introduced a severe bias to this meta-analysis. Another study, which investigated patients suffering from Rhinitis Sicca, was rejected because of the differing disease pattern. Thus, the meta-analysis was based on data from four unpublished studies. Of these, three studies included only adults, while one study investigated the efficacy of ectoine in children. Details on the integrated studies are shown in Table 1.

All studies were performed in ENT medical practices in Germany.

In total, 112 patients were included in the analyses comparing the symptom scores on Day 7 and at baseline (Day 1), while the meta-analysis based on the AUC comprised 213 participants. This difference was due to unbalanced numbers of patients in each group of comparison.

We performed the meta-analysis in line with a statement proposed by the international MOOSE group [26] about the conduct of meta-analyses of observational studies. Their recommendations concern the entire process of performing a meta-analysis — from describing background, search strategy, and methodology applied to presentation of results and discussion.

All data presented are based on the ITT analysis set of each study.

### 3.2. Bias

As with any systematic review or meta-analysis, biases may be present and limit the validity of the work. The main concern of this meta-analysis may be the quality of the included studies. In contrast to large systematic reviews, this work was mainly based on observational studies with no blinding of the patients or investigators. Since only one randomised, placebo controlled trial on children has been performed and published on this specific topic, the methodological concepts of the included trials do reach the evidence level IIb but not Ib. We addressed this “garbage in/garbage out” problem [27, 28] in performing a subgroup analysis on observational studies with adults. Apart from that no major conceptional differences between the studies were apparent, which minimises the risk concerning problems with uniformity (“apple-oranges problem”) [29]. Endpoints, nasal symptoms, measurements, and the study population were comparable. However, scaling systems in rating the symptom severity differed slightly and had to be transformed into a homogeneous scaling scheme. It is questionable whether this adaption leads to a loss of information or a shift of results. However, the tendency of whether symptoms were released or not is not biased by this approach. Furthermore, the variation of control groups may limit the validity of the meta-analysis. Ectoine was compared to four different control medications, since the major interest was about the efficacy of the active agent ectoine in comparison to general drugs prescribed. As already mentioned before, this meta-analysis was based on only small, unpublished clinical trials. Thus, one can speak of a very untypical publication bias with solely data from yet unpublished studies. Given the small number of included studies, we refrained from performing a funnel plot.

### 3.3. Development of Symptoms

**Figure 2:**
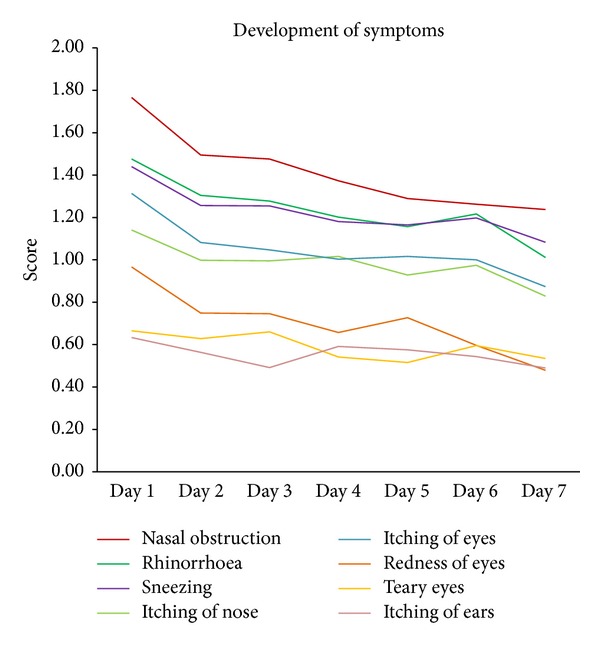
Development of symptoms.

Figure 2 illustrates the cumulative efficacy of ectoine-based products on both nasal and eye symptoms based on results from the included studies. The descending curve progression affirmed the positive effect of ectoine on rhinitis-related symptoms. At baseline, nasal obstruction presents the most predominant symptom of the allergic disease. After seven days of treatment, each symptom had improved to a mild level of discomfort (see Figure 2). The strongest decrease in nasal symptom severity was shown for rhinorrhoea and nasal obstruction, both being reduced by approximately 30%. For nasal obstruction, a symptom score of 1.77 at Day 1 decreased to a mean score of 1.24 and the symptom severity of rhinorrhoea eased from 1.48 to 1.01 after seven days of treatment with ectoine nasal spray.

According to the patients' diary entries, however, none of the symptoms was assessed as moderate or severe at baseline, but mild to moderate at the most. The rather mild assessment of symptoms at baseline limited the prospects of significant improvement. However, the apparent decrease in symptom severity suggests the efficacy of ectoine-based treatment.

### 3.4. Meta-Analyses as Comparison of Baseline (Day 1) to Day 7

The meta-analyses of individual symptoms that were conducted to determine the strength of effect after seven days of medication intake compared to baseline indicated the efficacy of ectoine. All nasal symptoms had significantly improved by Day 7 compared to Day 1.

**Figure 3:**
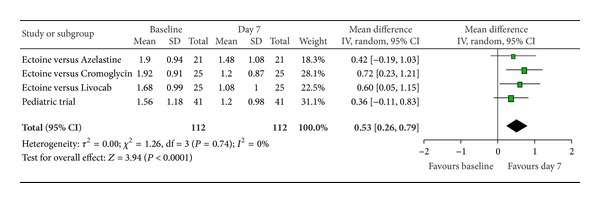
Nasal obstruction.

**Figure 4:**
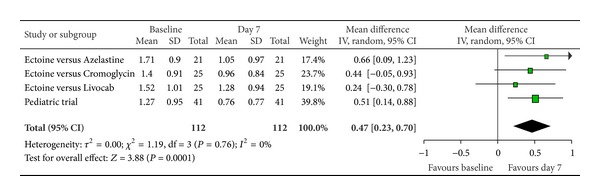
Rhinorrhoea.

**Figure 5:**
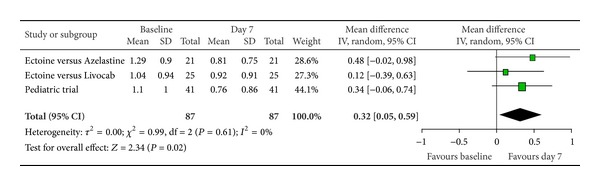
Nasal itching.

**Figure 6:**
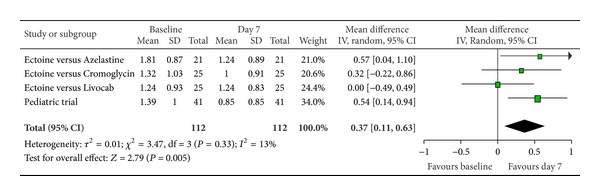
Sneezing.

According to Ferguson the effect size of improvement can be classified in three categories: 0–0.2 reflecting a small effect, 0.2–0.5 indicating a moderate effect, and 0.5–0.8 representing a strong effect [30]. Therefore, the improvement of the main nasal symptom “nasal obstruction” (Figure 3) with a size effect of 0.53 (±0.26) was evaluated as strong. Further nasal symptoms still showed significant moderate effects. The effect size for “rhinorrhoea” (Figure 4) was nearly as high with 0.47 (±0.24), “nasal itching (Figure 5) was calculated as 0.32 (±0.27), and for “sneezing” (Figure 6) the effect size was 0.37 (±0.26). *P* values of the overall effect (shown underneath each figure) demonstrate significance for all nasal symptoms: both “nasal obstruction” and “rhinorrhoea” were associated with *P* < 0.0001; the symptom “nasal itching” corresponds to *P* = 0.02; the *P* value for “sneezing” was calculated as *P* = 0.005.

**Figure 7:**
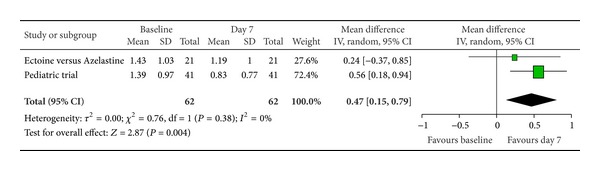
Itching of eyes.

**Figure 8:**
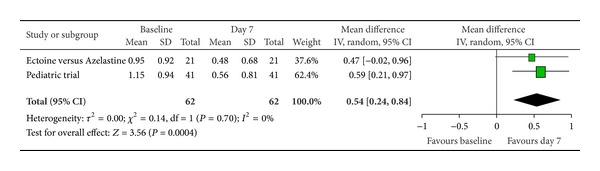
Redness of eyes.

**Figure 9:**
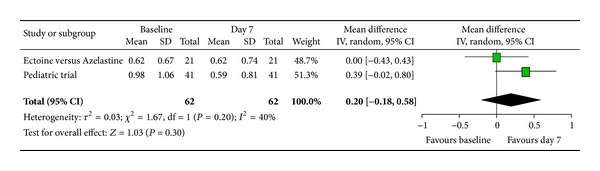
Teary eyes.

Furthermore, we pooled data from two studies that additionally used ectoine-based eye drops to investigate the effect of ectoine on eye symptoms. After seven days of treatment, “itching of eyes” (Figure 7) and “redness of eyes” (Figure 8) showed significant improvements compared to baseline. Both parameters improved by a moderate-to-strong effect size with 0.47 (±0.32) and 0.54 (±0.30), respectively. Only the reduction of symptom severity in “teary eyes” (Figure 9) was not statistically significant.

Throughout the analyses, the level of heterogeneity was low. As suggested by Higgins et al. [31], values for *I* ^2^ < 25% may express a low level of heterogeneity, although its categorisation and quantification are not that simple in general. Since *I* ^2^ was calculated to be in a range of 0% to 13% for most symptoms (apart from the symptom “teary eyes”), the heterogeneity across studies appears to be small.

### 3.5. Meta-Analyses of the Area under the Curve (AUC) Comparing Ectoine with Control Medication

**Figure 10:**
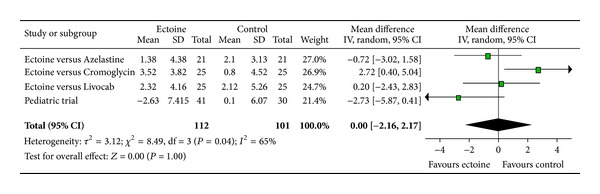
AUC nasal obstruction.

**Figure 11:**
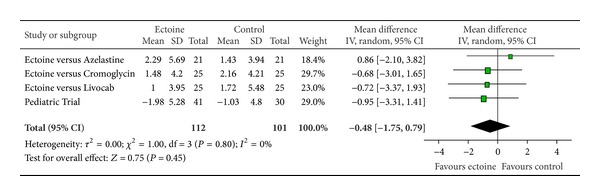
AUC rhinorrhoea.

**Figure 12:**
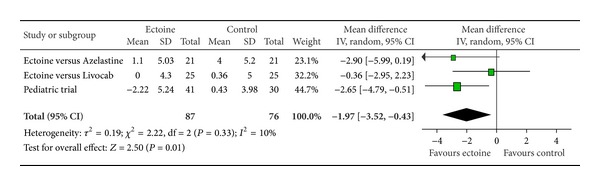
AUC nasal itching.

**Figure 13:**
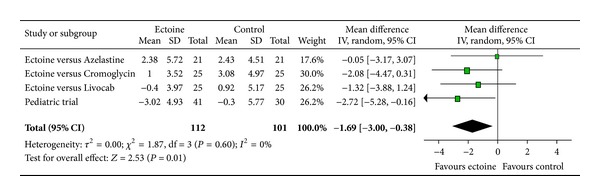
AUC sneezing.

The meta-analyses of AUC, comprising Day 1 to Day 7, evaluated the efficacy of ectoine treatment in comparison to placebo or to a standard medication (control) for allergic rhinitis. One study used azelastine nasal spray as comparator, in the second study a nasal spray based on cromoglicic acid served as control medication, the third study investigated ectoine nasal spray versus levocabastine (Livocab) with beclomethasone nasal spray, and the paediatric study was set up as a placebo-controlled trial. Although it is principally not recommended to pool data from studies with different control groups, the approach seemed appropriate since we were able to extract original data for each symptom individually. For all symptoms ectoine-containing nasal spray demonstrated similar or better efficacy when compared to controls. Effects were greatest for the symptoms “nasal itching” (−1.97 ± 1.54) (Figure 12) and “sneezing” (−1.69 ± 1.31) (Figure 13) which were associated with significant differences in favour of ectoine. For the remaining nasal symptoms “nasal obstruction” (Figure 10) and “rhinorrhoea” (Figure 11), the meta-analysis revealed that ectoine is similarly effective compared to the control drugs.

**Figure 14:**
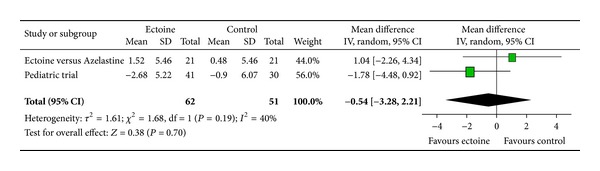
AUC itching of eyes.

**Figure 15:**
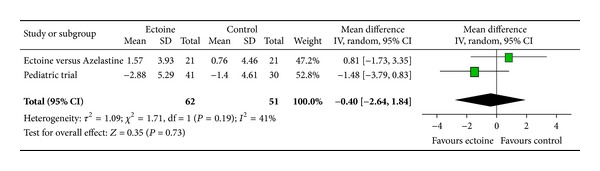
AUC redness of eyes.

**Figure 16:**
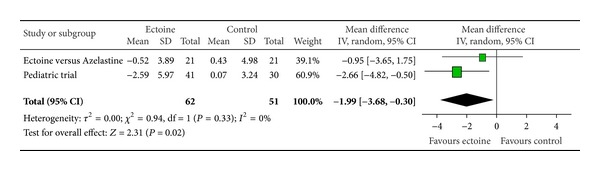
AUC teary eyes.

Since only two studies investigated the effect of the medication on eye symptoms, we pooled data from these two studies (ectoine versus azelastine and paediatric trial) to evaluate the effect of ectoine on the eyes. The analysis reveals that the symptom “teary eyes” (Figure 16) was significantly improved (*P* = 0.02) by the ectoine-containing nasal spray and eye drops with an effect size of −1.99 (±1.69). The symptoms “itching of eyes” (Figure 14) and “redness of eyes” (Figure 15) both tended slightly towards the ectoine products with effect sizes of −0.54 (±2.75) and −0.40 (±2.24), respectively. However, no statistical significance was reached here.

## 4. Subgroup Analyses

Subgroup analyses were performed for the two main allergic rhinitis symptoms of nasal obstruction and rhinorrhoea in order to evaluate the effect of ectoine in the specific group of adults with allergic rhinitis. Three studies with a total of 71 patients were included, whereby the level of heterogeneity decreased to 0%. Again, the three control groups of the integrated studies (azelastine, levocabastine/beclomethasone, and cromoglicic acid) were pooled into one control group versus ectoine nasal spray.

**Figure 17:**
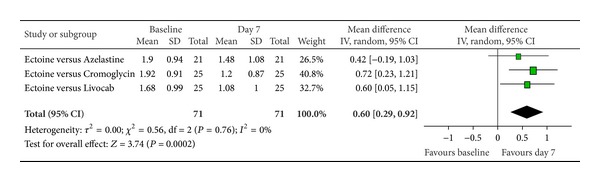
Subgroup nasal obstruction.

**Figure 18:**
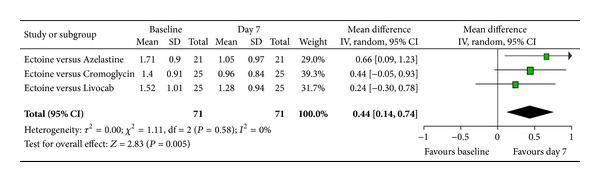
Subgroup rhinorrhoea.

The subgroup analyses clearly emphasised the positive effect of ectoine nasal spray after seven days of treatment. Since each individual study consistently expressed the efficacy of ectoine, the overall pooled result for both nasal obstruction and rhinorrhoea was significant in favour of a seven-day medication intake. The corresponding *P* values were *P* = 0.0002 for the effect on nasal obstruction and *P* = 0.005 for rhinorrhoea. With a total effect of 0.6 (±0.31) for nasal obstruction (Figure 17), the efficacy of the ectoine-based nasal spray after seven days was associated with a strong effect size. Similarly, the total effect size of 0.44 (±0.31) for rhinorrhoea (Figure 18) signified a moderate effect of ectoine nasal spray. Therefore, the subgroup analyses confirmed the positive effect of ectoine nasal spray in alleviating the predominant symptoms of nasal obstruction and rhinorrhoea in adult patients with allergic rhinitis.

## 5. Discussion

This meta-analysis served to compare the treatment of allergic rhinitis with ectoine-containing nasal spray and eye drops to traditional treatment agents (antihistamine, glucocorticoid, and cromoglicic acid) or placebo treatment.

The meta-analysis involving ectoine nasal spray in the categories of baseline comparison and AUC determined a reduction in symptom severity for all relevant rhinitis symptoms. An especially strong effect was shown for the symptom of nasal congestion, which dropped significantly by 29.94% after seven days of treatment. According to the classification scheme developed by Ferguson [30], the improvement of nasal obstruction was categorised as strong, while further nasal symptoms such as rhinorrhoea, nasal itching, and sneezing were still associated with a significant improvement of moderate effect size. Likewise, significant improvements with a strong and moderate effect size were also demonstrated for nasal obstruction and rhinorrhoea in the subgroup analysis of adult SAR patients.

While ectoine-based products were shown to act significantly more effective than the control medications in easing the symptom severity of nasal itching, sneezing, and teary eyes, results for the remaining symptoms still confirmed a similar potency of ectoine nasal spray compared to standard medication.

Two studies during which ectoine-containing eye drops were used additionally to the application of ectoine nasal spray demonstrated improvement of ocular symptoms. Here, a strong size effect was shown in reducing red eyes and moderate size effect in reduction of itching eyes. Likewise, the analysis of accumulated effects revealed a significant improvement for the symptom “teary eyes” in the ectoine group. These results indicate a positive influence of ectoine eye drops on ocular symptoms in seasonal allergic rhinitis. However, further studies are needed to confirm these findings as the possibility that the effect may be explained by the inhibition of the naso-ocular reflex, as it has been suggested in studies with intranasal steroids [32], cannot be excluded based on the current results.

In this meta-analysis, we compared the efficacy of ectoine to three effective, currently guideline-recommended medications, such as the second-generation antihistamine azelastine, the glucocorticoid combination levocabastine/beclomethasone, and the classical cromoglicic acid. The comparison attested the equivalence of ectoine nasal spray to these products. Thus, the ectoine-based products can be regarded as noninferior to topical antihistamines, the intranasal glucocorticosteroid combination levocabastine/beclomethasone, or nasal mast cell stabilisers for the treatment of rhinitis symptoms.

The results of this meta-analysis are promising and further supported by the safety profile of products containing ectoine [20, 33]. Clinical studies have shown that treatment with ectoine results in very few adverse events (frequency comparable to placebo) and virtually no safety concerns [20, 33, 34]. In contrast, the traditional drugs are still associated with side effects, warranting the search for alternative treatments. Thus, particularly antihistamines, such as azelastine, even in nasal spray form, continue to cause sedation/somnolence and nasal burning occasionally (Astelin patient information). Moreover, nasal steroids can also have various adverse effects. For example, the patient information for glucocorticoid nasal spray, for example, fluticasone furoate (Veramyst), warns about possible ocular side effects including glaucoma, cataracts, and increased intraocular pressure. While—in isolated cases—growth retardation has been associated with beclomethasone treatment [35], nasal spray, and eye drops containing ectoine offer adequate relief from allergy symptoms without these added risks. However, those infrequent side effects should not be overestimated, and newer drug formulations show fewer adverse reactions than the earlier agents. Still, the absence of safety concerns makes ectoine-based products particularly interesting candidates for the treatment of allergic rhinitis in children. Since the application of corticosteroids in children has raised some concerns regarding impaired growth and abnormal development, ectoine may provide a safe and convenient alternative for physicians and parents worried about treating their allergic children with pharmaceutical products that have potential harmful side effects. However, the safety of ectoine nasal spray needs to be further investigated in future studies to confirm the safety profile of the product.

The mode of action of ectoine-based products in preventing and relieving allergic symptoms is based on the physical interaction of ectoine with water and the resulting effects on the membrane of the tissue treated. Stabilisation of cell membranes with consequent enhancement of the tissues' barrier function may reduce the allergen-membrane interactions and inflammation which usually cause ocular, nasal, and nonnasal symptoms in patients with allergic rhinitis.

There is one constraint to this meta-analysis: upon inclusion, patients had mostly mild symptoms. Hence, no large improvements could be expected from a one- to two-week course of treatment. Considering these baseline values, the verified improvement can indeed be interpreted as convincing. Future studies including patients with more severe baseline symptoms would be needed to further investigate the effectiveness of ectoine treatment in rhinitis patients. A further limitation concerns the methodology, since only data from unpublished studies are included in this meta-analysis. The included study data have not been published to date, since the number of participants in each trial was too small to show interesting results. Nowadays, large randomised controlled trials with more than 250 patients per treatment group are commonly required to be considered for publication [21, 22]. Likewise, published noninterventional studies are usually performed with numbers larger than 1000 patients to be powered adequately [36, 37]. To date, no publications investigating ectoine as a nasal spray ingredient exist.

## 6. Conclusion

Taken together, this meta-analysis demonstrated that the application of ectoine-based nasal spray and eye drops improves symptoms of allergic rhinitis and rhinoconjunctivitis. This easy-to-apply, well-tolerated, naturally-based nasal and ocular treatment, which has no unpleasant taste and virtually no side effects, effectively reduces allergic rhinitis symptoms and represents an exciting alternative for rhinoconjunctivitis sufferers.

## Figures and Tables

**Table 1:** Description of included studies.

Study ID	Indication of study	Comparator	Study design	*N* (Ectoine and control)	Inclusion criteria/exclusion criteria	Duration and dosage	Outcome Parameter	Rating scales
Ectoine versus azelastine, 2010	Allergic rhinitis and conjunctivitis	Azelastine nasal spray and azelastine eye drops	Observational	Ectoine: 21 Control: 21	AR proven by a diagnostic tool or by an allergist Moderate-to-strong nasal symptoms and at least mild eye symptoms at inclusion Signed informed consent Exclusion: allergy to ectoine or azelastine, pregnancy, operation of nose	1 week, ectoine: 4 times daily Azelastine: 2 times daily	Primary: Nasal congestion, rhinorrhoea, sneezing, nasal itching, itching of eyes, itching of palate, teary eyes, conjunctivitis. Secondary: Efficacy, safety.	8-point scale

Ectoine versus cromoglicic acid 2009	Allergic rhinitis	Cromoglicic nasal spray	Observational, cross-over trial	Ectoine: 25 Control: 25	Inclusion: AR proven by skin prick test or by an allergist Moderate-to-strong nasal symptoms at inclusion Signed informed consent Exclusion: allergy to ectoine or cromoglicic acid, pregnancy, operation of nose	1 week, ectoine: 5 times daily Cromoglicic acid: 4 times daily	Primary: Nasal congestion, rhinorrhoea, sneezing. Secondary: Itching of eyes, itching of palate, teary eyes, conjunctivitis, nasal concha hyperplasia, efficacy, safety.	8-point scale

Ectoine versus Livocab, 2011	Seasonal allergic rhinitis	Levocabastine with beclomethasone 0.05% (nasal spray)	Observational	Ectoine: 25 Control: 25	Inclusion: 18–70 years Signed informed consent	2 weeks, intake of medication according to prescribing information	Primary: Total Nasal Symptom Score as sum of nasal congestion, rhinorrhoea, sneezing and nasal itching. Secondary: Itching of palate and ears, efficacy, safety.	4-point scale

Paediatric trial Ectoine versus placebo, 2011	Seasonal allergic rhinitis	Placebo nasal spray and eye drops	RCT	Ectoine: 41 Control: 30	Inclusion: 5–17 years Seasonal AR, general good health status, free of any concomitant conditions that could interfere with conduct of study, sum of TNSS >5, sum of TOSS >3 Signed informed consent (also by parents)	2 weeks Nasal spray: 1 puff per nostril 3 times daily Eye drops: 3 times daily	Primary: Safety Secondary: Efficacy assessment, Total Nasal Symptom Score as sum of runny nose, itchy nose, nasal congestion, sneezing; Total Ocular Symptom Score as sum of itchy eyes, red eyes, watery eyes.	4-point scale
